# Native RNA or cDNA Sequencing for Transcriptomic Analysis: A Case Study on *Saccharomyces cerevisiae*

**DOI:** 10.3389/fbioe.2022.842299

**Published:** 2022-04-12

**Authors:** Thidathip Wongsurawat, Piroon Jenjaroenpun, Visanu Wanchai, Intawat Nookaew

**Affiliations:** ^1^ Division of Bioinformatics and Data Management for Research, Research Group and Research Network Division, Research Department, Faculty of Medicine Siriraj Hospital, Mahidol University, Bangkok, Thailand; ^2^ Department of Biomedical Informatics, College of Medicine, University of Arkansas for Medical Sciences, Little Rock, AR, United States

**Keywords:** direct RNA sequencing, direct cDNA sequencing, differential gene expression, RNA modification, 3′ bias, native sequence, yeast, long-read technology

## Abstract

Direct sequencing of single molecules through nanopores allows for accurate quantification and full-length characterization of native RNA or complementary DNA (cDNA) without amplification. Both nanopore-based native RNA and cDNA approaches involve complex transcriptome procedures at a lower cost. However, there are several differences between the two approaches. In this study, we perform matched native RNA sequencing and cDNA sequencing to enable relevant comparisons and evaluation. Using *Saccharomyces cerevisiae*, a eukaryotic model organism widely used in industrial biotechnology, two different growing conditions are considered for comparison, including the poly-A messenger RNA isolated from yeast cells grown in minimum media under respirofermentative conditions supplemented with glucose (glucose growth conditions) and from cells that had shifted to ethanol as a carbon source (ethanol growth conditions). Library preparation for direct RNA sequencing is shorter than that for direct cDNA sequencing. The sequence characteristics of the two methods were different, such as sequence yields, quality score of reads, read length distribution, and mapped on reference ability of reads. However, differential gene expression analyses derived from the two approaches are comparable. The unique feature of direct RNA sequencing is RNA modification; we found that the RNA modification at the 5′ end of a transcript was underestimated due to the 3′ bias behavior of the direct RNA sequencing. Our comprehensive evaluation from this work could help researchers make informed choices when selecting an appropriate long-read sequencing method for understanding gene functions, pathways, and detailed functional characterization.

## Introduction

The RNA sequencing (RNA-seq) method is now routinely used to explore a collection of all the gene readouts present in a cell or its transcriptome. Transcriptomic changes are a result of biological differences, making RNA-seq an exceptional opportunity to explore global regulatory networks in cells, tissues, organisms, and diseases. The most common RNA-seq methodology currently offered by next-generation sequencing (NGS) platforms (i.e., Illumina, Ion Torrent, and MGI) produces hundreds of millions of short-read sequences in the range of 100–600 base pairs. The short-read RNA-seq power provides not only large data output but also high accuracy in base calling. The short-read RNA-seq, therefore, is now a core component of research in nearly all biological fields (Wang et al., 2009; Ozsolak and Milos, 2011).

Despite dominant position of NGS in transcriptomics, short-read RNA-seq has been poorly suited for transcriptome assembly, novel splice isoform discovery, and novel gene detection. Because most eukaryotic messenger RNA (mRNA) transcripts are 1–2 kb in length (Harrow et al., 2012), no matter how deeply they are sequenced, the short-reads have to be computationally assembled into full-length transcripts. Although this is performed using powerful algorithms, they often fail to resolve complex transcript isoforms expressed by the same gene. Because of these limitations, if the reads are too short, the predicting transcripts have high false-positive rates. In addition to the problem of isoform detection, various sources of bias inherent to short-read RNA-seq have also been identified, such as GC-content (Benjamini and Speed, 2012), PCR amplification (Hansen et al., 2010), and transcript quantification (Li et al., 2010).

The rise of long-read technologies now opens the possibility to overcome those limitations and biases. Long-read RNA-seq captures a full-length transcript within a single read, thereby allowing accurate transcript annotation and enabling a comprehensive view of the transcriptome. Sequencing prokaryotic transcriptomes using the long-read technology reveals complex operon structures, which provide an important resource for functional annotation (Yan et al., 2018). Currently, the most widely used platforms are Pacific Biosciences (PacBio) and Oxford Nanopore Technologies (ONT). With the read lengths achieved with PacBio (∼15 kb) and with ONT (>30 kb), both surpass lengths of most transcripts (Harrow et al., 2012; Oikonomopoulos et al., 2020; Udaondo et al., 2021). However, with ONT, if native RNA can be directly sequenced RNA (dRNA-seq) without PCR amplification, then amplification biases are eliminated (Workman et al., 2019). The direct sequencing feature permits detection of RNA base modifications, such as N6-methyladenine (m6A), which has been linked to human obesity and cancer (Mortazavi et al., 2008). In addition, ONT is a more cost-effective method than PacBio in terms of machine cost and number of bases per 1,000 USD (Byrne et al., 2019).

Using dRNA-seq (Jenjaroenpun et al., 2018), we recently showed a transcriptional landscape analysis of the *Saccharomyces cerevisiae* strain, CEN.PK113-7D, a yeast strain that is used extensively in academic and industrial research. We determined transcriptomic profiling under two different growth conditions (diauxic growth). Approximately 70% of the reads corresponded to full-length transcripts. Some full-length transcripts over 5 kb were also detected and mapped. In addition, identification of polyadenylated non-coding RNAs (i.e., ribosomal RNA, telomerase RNA, and long non-coding RNA) is allowed using this sequencing protocol (Jenjaroenpun et al., 2018).

After releasing the dRNA-seq approach, a direct cDNA sequencing (dcDNA-seq) protocol was subsequently released by ONT. The latter protocol is also PCR-free and carries out large complex whole-genome analysis at lower cost. However, there are several differences between the two approaches; notably, RNA and DNA sequencing speeds are different (typically ∼85 bp per second for RNA (Garalde et al., 2018) vs. 450 bp per second for DNA (Rang et al., 2018)). Charlotte Soneson et al. applied matched dRNA-seq and dcDNA-seq to samples from human cell lines. The study showed the potential advantages that the dRNA-seq brings over the short-read sequencing and that it could be an important addition to the mammalian transcriptomic toolbox (Soneson et al., 2019). In addition to human cells, dRNA-seq was successfully used to study the transcriptomic characteristics in insect species, which was characterized by large and repetitive genomes (Jiang et al., 2019). Therefore, in this proposed work, we will apply ONT dcDNA-seq to RNA samples extracted from the *Saccharomyces cerevisiae* strain, CEN.PK113-7D. Then, we will perform a detailed comparison of reads from dRNA-seq from the perspective of RNA modification. Our comprehensive evaluation from this proposed work will help researchers make informed choices when selecting an appropriate long-read sequencing method for understanding gene functions, pathways, and detailed functional characterization for further development of yeast biotechnology.

## Materials and Methods

### Cell Culture and RNA Purification

The details of cell growth and RNA purification were previously described (Jenjaroenpun et al., 2018). In brief, the *Saccharomyces cerevisiae* strain CEN.PK113-7D was cultured in glucose-limited conditions in the defined media with an initial glucose concentration of 20 g/L. At the mid-log growth on glucose and oxidative growth on ethanol, the cells were collected and quickly frozen using liquid nitrogen and stored at −0°C. RNA was then extracted from the frozen cells using the RNeasy Mini Kit (Qiagen) following the manufacturer’s protocol.

### Library Preparation, dcDNA-Seq, and dRNA-Seq by ONT

**FIGURE 1:**
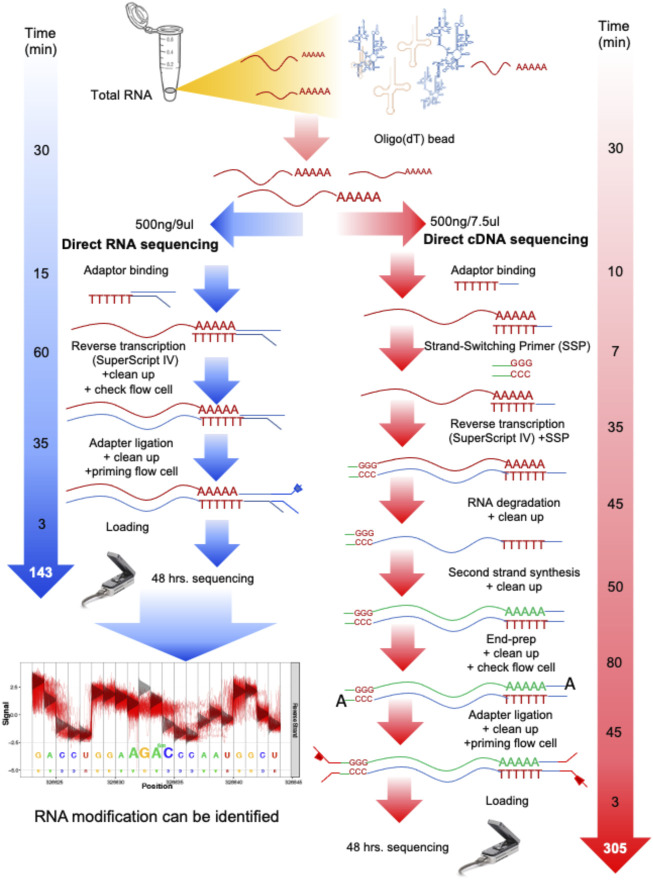
Overall process and time spent on direct RNA sequencing (dRNA-seq) and direct cDNA sequencing (dcDNA-seq) using Oxford Nanopore Technologies (ONT). Both sequencing methods are used to prepare any RNA with a 3′ poly-A tail, such as eukaryotic mRNA or viral RNA with a poly-A tail. For the dRNA-seq, the native RNA passes through the nanopore, therefore the read length reflects the length of the RNA molecules, and the identification of RNA modification is allowed. The dcDNA-seq and poly-A RNA were used to prepare cDNA copies for sequencing, which takes more steps and a longer time for library preparation. However, the speed of cDNA sequencing is faster than that of RNA sequencing. Thus, the dcDNA-seq usually provides a higher yield (more bases).

In this work, we started from aliquoted poly-A RNA used in our previous work (Jenjaroenpun et al., 2018). Briefly, total yeast RNA (∼24 μg) obtained from three biological replicates of each condition (glucose and ethanol) was previously enriched for poly-A RNA by means of oligo(dT) beads, collected, and stored at −80°C. For dcDNA-seq, the starting amount of poly-A RNA of 500 ng was used as the RNA input. The dcDNA library was produced using the Direct cDNA Sequencing Kit, SQK-DCS108 Kit (ONT). The RNA was converted to double-stranded DNA, and then strand-switching and adaptor ligation were performed (Figure 1). The library was loaded onto a flow cell (R9.5/FLO-MIN107 flow cell) for sequencing using a MinION Mk1B for a 48-h sequencing run.

For dRNA-seq, the SQK-RNA001 Kit was used (ONT), and Superscript IV Reverse Transcriptase (ThermoFisher) was applied for the RNA stabilization step by formation of DNA–RNA hybrids through reverse transcription. After this, the motor protein was attached specifically to the RNA strands (Figure 1). Each library was loaded onto a flow cell for a 48-h sequencing run. Direct sequencing of the poly-A RNA (dRNA) was performed on a single R9.5/FLO-MIN107 flow cell.

### Bioinformatics and Statistical Analysis

#### Data Processing and Mapping of Reads

The ONT raw data (.fast5 files) generated by MinKnow software, version 1.7.14 (ONT), were converted to basecalled fastq files using the local-based software Guppy, version 3.4.5 (ONT). This step automatically classifies failed and passed reads based on a specific cut-off for mean quality scores of 7, and only reads of >200 bases were included. The ONT reads (in standard fastq format) were aligned to the yeast S288c version R64 reference sequences, downloaded from SGD database, using Minimap2 (Li, 2018) to generate a BAM file (a binary version of a Sequence Alignment Map [SAM] file).

#### Evaluation of mRNA Sequencing Characteristics

The dRNA reads were converted to DNA sequences, and reverse complement sequences of dcDNA reads were generated before alignments. For analysis of mapping results of yeast, we used SAMtools, version 1.6 (Li et al., 2009), to investigate the BAM files and to classify sequence reads into categories of mapped, unmapped, chimeric, and other reads based on standard Concise Idiosyncratic Gapped Alignment Report (CIGAR) string information (a compressed representation of an alignment).

#### Differential Gene Expression Evaluation

We followed the workflow to analyze differential gene expression of yeast transcripts as previously described (Jenjaroenpun et al., 2018). In brief, the read count table of individual transcripts for the dcDNA and dRNA sequences was generated using multicov from Bedtools version 2 (Quinlan and Hall, 2010). We then used the DESeq2 package (Love et al., 2014) to calculate adjusted *p*-values of individual transcripts between the two compared growth conditions. We considered that the gene that has adjusted *p*-value < 0.001 was differentially expressed. Consequently, functional gene enrichment analysis based on Gene Ontology (GO) annotation was performed using the Platform for Integrative Analysis of Omics data (PIANO) package (Varemo et al., 2013).

#### Inferring RNA Modification From Sequencing Error Profile

We inferred the RNA modifications of dRNA sequences using our developed epitranscriptional/epigenomical landscape inferring from glitches of ONT signals (ELIGOS) software (Nookaew et al., 2020; Jenjaroenpun et al., 2021; Boysen and Nookaew, 2022) with two approaches. First, profiling of RNA modifications of the individual growth condition was performed by comparing the error at specific base (ESB) with the RNA background error model (rBEM). Second, differential RNA modification was performed by direct comparison of ESB between yeast cells grown on glucose and ethanol. As the study has three biological replicates, we employed Cochran–Mantel–Haenszel statistical test for comparisons. We developed an additional function “multi_samples_test” for Cochran–Mantel–Haenszel statistical test with default parameters and updated it in the ELIGOS software (Jenjaroenpun et al., 2021).

#### RNA Structure Prediction Using ShaKer and RNAplfold

To examine the secondary structure of RNA, we used a selective 2′-hydroxyl acylation analyzed by primer extension (SHAPE) prediction using the graph kernel (ShaKer) tool (Mautner et al., 2019). This *in silico* approach provides an advantage over other SHAPE prediction tools, which require manually curated reference RNA structures. We implemented an in-house python script using libraries from the ShaKer tool for training a SHAPE model and to predict RNA structure and accessibility. A general model of SHAPE reactivity was trained on an experimentally determined SHAPE dataset provided in the ShaKer repository. Then, the predicted SHAPE model was used to support the prediction of structure and accessibility on each nucleotide of a given RNA sequence using RNAplfold (Lorenz et al., 2016). The score form ShaKer, derived from the default parameters, was used to determine the correlation with RNA modification sites based on odds ratios obtained from ELIGOS (Jenjaroenpun et al., 2021).

#### Genomic Locations of Loci and Transcript Comparison

The relative location of considered loci with reference to gene position was compared using Bedtools version 2 (Quinlan and Hall, 2010).

The parameters and commands used are summarized in Supplementary Note S1.

## Results

### Library Preparation and Sequencing of dcDNA-Seq and dRNA-Seq

To compare dcDNA-seq vs. dRNA-seq, the poly-A mRNA isolated from yeast cells grown in minimum media supplemented with glucose and from cells that had switched to ethanol as a carbon source were aliquoted and used as the input of the two sequencing strategies to rescued batch effect. For each condition, three biological replicates were analyzed. The sequencing workflow of the two sequencing strategies is summarized in Figure 1. The processing time of dRNA-seq is approximately 135 min, which is about half of the dcDNA-seq time, due to the minimal manipulation of the mRNA molecules, and results in only a four-step procedure of library preparations. The dcDNA-seq library preparation requires approximately 305 min for seven steps for the first and second strands of cDNA synthesis before sequencing. The sequencing for the two strategies was performed with the same time of 48 h.

### Sequence Characteristics of dcDNA-Seq and dRNA-Seq

**FIGURE 2:**
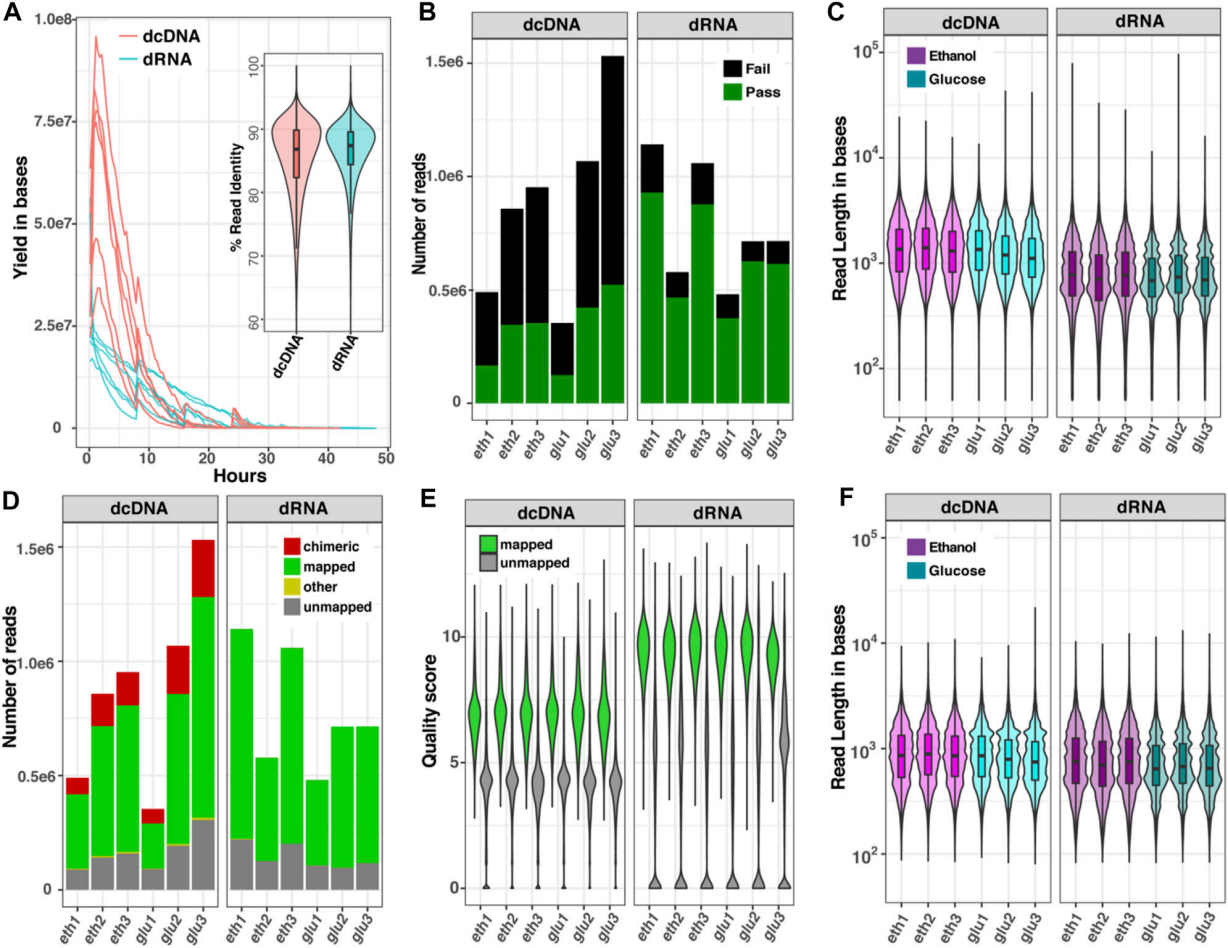
Comparison of read characteristics for six datasets of yeast RNA sequenced as dcDNA or dRNA. **(A)** Sequence yields per hour and violin boxplot of %read identity; **(B)** numbers of reads that passed (green) or failed (black) the quality score of 7 by Guppy software; **(C)** read length distribution of all reads combined (passed plus failed); **(D)** numbers of all reads that could be mapped to a reference genome; **(E)** quality score distribution of mapped and unmapped reads; and **(F)** read length distribution of the reads after removal of chimeric sequences. Data are shown for glucose-grown cells (glu) and for glucose-deprived cells (eth).

The differences in read characteristics obtained from dcDNA-seq and dRNA-seq for the two transcriptomes are summarized in Figure 2. The sequence yield obtained per hour on the ONT flow cells (Figure 2A) was higher for dcDNA than for dRNA due to the different motor proteins that control the rate of molecules passing through the nanopores [450 bases per second (b/s) for DNA and 80 b/s for RNA sequencing]. The average percent identities of both dcDNA and dRNA reads were comparable, around 88% (violin plot, Figure 2A). The base-calling step using Guppy software automatically classifies reads to pass or fail based on a specific cut-off. As seen in Figure 2B, on average 85% of the total dRNA reads, but only 50% of dcDNA reads, passed the default threshold of 7. The length of all reads combined (passed plus failed) indicated that the dcDNA reads were slightly longer than the obtained dRNA reads (Figure 2C).

To explain the surprisingly high fraction of failed reads obtained with dcDNA, we re-evaluated the quality of total reads (passed plus failed) by aligning both dcDNA and dRNA reads onto a reference genome. As presented in Figure 2D, 61–67% of the dcDNA reads could be mapped, while 80–86% of the dRNA reads mapped to the reference genome. Of note was the relatively high fraction of chimeras in dcDNA (15–20%), while the fraction of unmapped reads (∼15%) did not significantly differ (*p*-value > 0.05) between dcDNA and dRNA sequences. Furthermore, the read quality score distribution of total reads differed between dcDNA and dRNA reads (Figure 2E), with higher scores obtained for dRNA reads. Typically, we get no strand bias from ONT DNA sequencing; however, we found that the dcDNA sequencing result had strong one-strand bias of reads derived from first-strand synthesis (Supplementary Figure S1). This indicated low yield of second-strand synthesis when construct cDNA by a strand-switch reaction in yeast influenced the quality of dcDNA sequences. When the read length distribution was compared after removal of chimeric sequences from the dcDNA reads, this resulted in a comparable read length distribution for both sequencing strategies (Figure 2F).

### Comparison of Differential Gene Expression by dcDNA-Seq and dRNA-Seq

**FIGURE 3:**
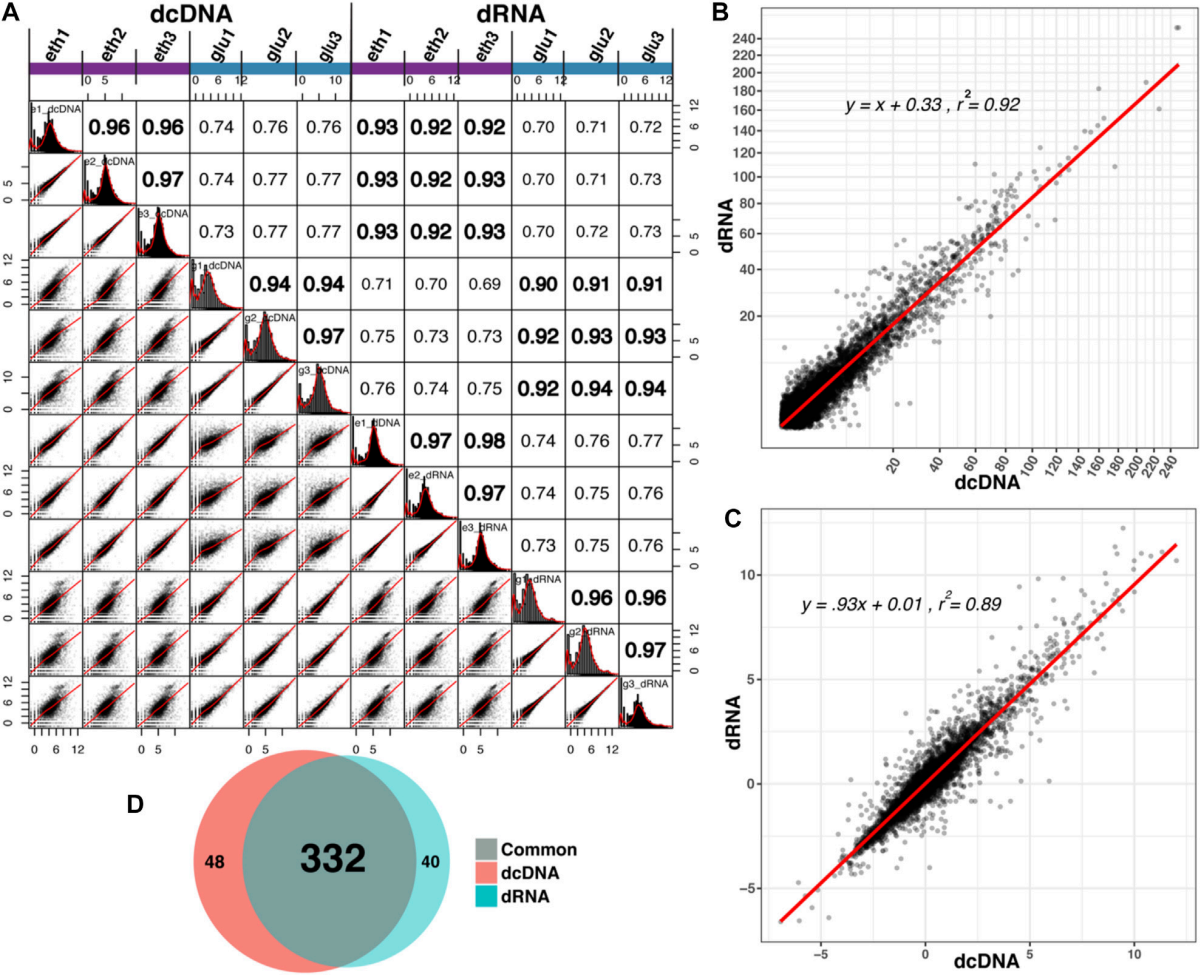
Comparison of transcript abundances based on dcDNA-seq and dRNA-seq. **(A)** Combined scatter plot and correlation matrix. **(B,C)** Scatter plots showing the correlation of statistical values between all individual transcripts combined as identified by dcDNA and dRNA based on adjusted *p*-values **(B)** and on observed mean log2fold changes **(C)** derived from three biological replicates. **(D)** Venn diagram of GO terms identified in dcDNA and dRNA datasets.

**FIGURE 4:**
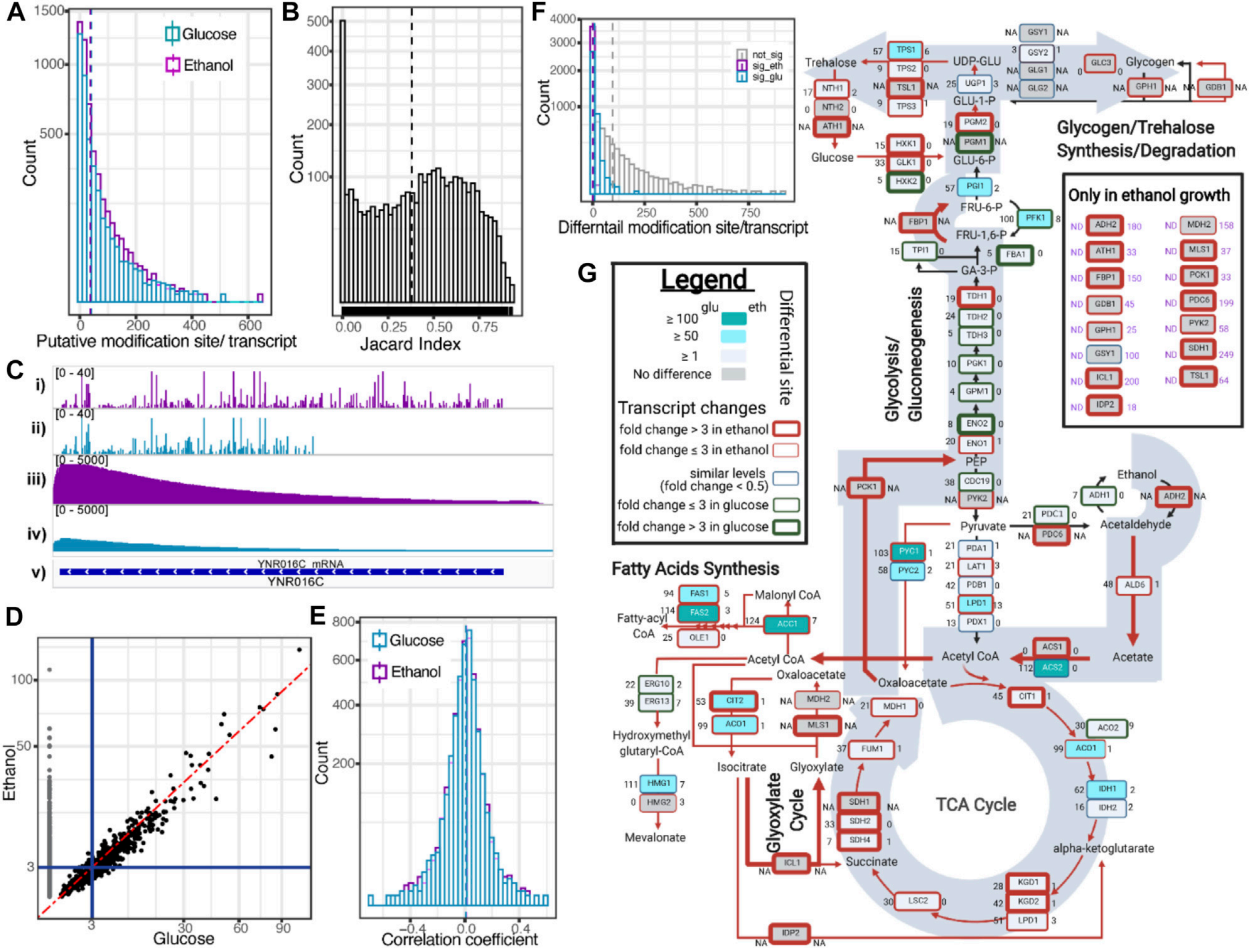
Capturing mRNA modification inferred from dRNA sequences. **(A)** Histogram plot shows putative modification site per transcript identified by ELIGOS. Dashed lines show the mean value. **(B)** Histogram plot shows Jaccard’s indexes representing degree of commonality of putative modification sites of individual transcripts between yeast grown in glucose and ethanol. Dashed line shows the mean value. **(C)** IGV snapshot of *ACC1* contains the highest number of putative modification sites and shows the comparison of identified putative modification sites of yeast grown in ethanol (i) and glucose (ii) with sequencing depth coverage along the transcript length (ethanol (iii) and glucose (iv)). The *ACC1* transcript region is shown in lane (v). **(D)** Scatter plot comparing odd ratios indicates the probability of RNA modifications along the *ACC1* transcript between yeast growth on glucose and ethanol. The black dots show the odd ratios that can be computed in both conditions. The gray dots show the odds ratios that can be computed only in ethanol growth. Red dashed line is a diagonal line. **(E)** Histogram plot showing Pearson’s correlation coefficient between odds ratios and the predicted SHAPE score. Dashed lines show the mean value. **(F)** Summary of differential modification sites per transcript between yeast growth on ethanol and glucose. The significant modification sites of ethanol and glucose growth are shown in magenta and cyan, respectively. The non-significant sites are shown in gray. **(G)** Summary of differential RNA modification in the central metabolic pathway that is known to be transcriptionally regulated between growth on glucose and ethanol. All *y*-axes of histogram plots are square-root scale. All magenta and cyan colors represent results of ethanol and glucose, respectively.

The read counts of individual transcripts derived from the two different templates (DNA and RNA) were compared by scatter plots, and a correlation matrix was constructed (Figure 4A). Within the same template, replicate experiments produced satisfying correlation coefficients (*r* = 0.96 on average, range: 0.94–0.98), while on average, *r* = 0.92 (range: 0.90–0.94) was obtained when dcDNA and dRNA sequences were compared for the same growth conduction. We recently demonstrated that the negative binomial statistic is a valid approach to analyze dRNA-seq data (Jenjaroenpun et al., 2018); here, we applied that method to compare the adjusted *p*-values (Figure 3B) and the observed mean log2fold changes (Figure 3C). Even though the sequencing depth across the biological replicates varied, the results of both sequencing methods strongly correlated for transcriptomes that were obtained from cells grown under the same condition. Furthermore, biological functional enrichment was analyzed using GO based on the dcDNA-seq and dRNA-seq data; the results were found to be highly consistent, as 332 GO terms were identified in both datasets, and only 48 GO terms were uniquely present in dcDNA-seq and 40 GO terms in dRNA-seq data (Figure 3D). The previously published conclusions on differential gene expression between the two compared culture conditions (Jenjaroenpun et al., 2018) did not change for the transcriptome sequencing data obtained from either dcDNA-seq or dRNA-seq.

### Inferred RNA Modifications From Native RNA Sequences

We performed the RNA modification profiling using ELIGOS software by comparing the ESB of dRNA sequences with rBEM. Based on the three biological replicates of yeast grown on glucose and ethanol, we identified 134,980 sites of putative RNA modification with glucose and 192,240 sites with ethanol using the cut-off of odd ratios ≥ 3 and adjPval < 1e-50. The distribution of the identified sites on the individual transcripts is shown in Figure 4A. The distribution of putative RNA modification sites per transcript of the yeast growth was quite similar, with a median of 17 sites per transcript with glucose and 20 with ethanol.

We further evaluated whether the identified putative RNA modification sites between the two conditions are common or not. We calculated Jaccard’s index between the two conditions of the identified sites of each individual transcript. We found that the distribution of Jaccard’s index is close to uniform distribution as shown in Figure 4B, indicating a random correlation. Interestingly, the highest frequency (642 sites in growth on ethanol) of putative RNA modifications sites was found on the transcript of a very important gene, encoding acetyl-CoA carboxylase (*ACC1*), which is the rate-limiting step enzyme of fatty acid biosynthesis. We further investigated the transcript in detail through the Integrative Genomics Viewer (IGV) browser (Figure 4C). The number of identified putative RNA modification sites of growth on glucose was 333, which is almost half of that grown on ethanol. It is clearly seen that there is no identified site that passed the statistical cut-off toward the 5′ end of the transcript (Figure 4C, track ii). The expression level of *ACC1* in ethanol growth condition is much higher than glucose growth as shown in the read coverage plots (Figure 4C, tracks iii and iv). The strong 3′ bias of dRNA reads was clearly observed, which could explain the missed identification of putative RNA modification near the 5′ end of the transcript, which has much fewer dRNA reads that were aligned, resulting in higher adjPval than the cut-off. Next, we created a scatter plot to compare the calculated odd ratios of the *ACC1* transcript between the two growth conditions, as shown in Figure 4D, and found a strong linear relationship. This indicated that the low sequencing depth on the 5′ end of dRNA sequencing will impact the confidence level of RNA modification identification (Figure 4D, gray dots), indicating an odds ratio that is too low in yeast grown in glucose.

The secondary structure of RNA plays important roles in the function of RNA molecules (Kertesz et al., 2010), and can be accurately probed by the SHAPE method (Wilkinson et al., 2006; Poulsen et al., 2015). Recently, a developed bioinformatic software, ShaKer, provided an accurate prediction of SHAPE using a graph kernel approach (Mautner et al., 2019). The accessible sites of the RNA molecule, such as on the loop, which has a high SHAPE score (Busan et al., 2019), are the frequently targeted sites for RNA modification of transfer RNA molecules (Han and Phizicky, 2018). We then compared the SHAPE scores obtained from ShaKer and the calculated odds ratios obtained from ELIGOS of individual transcripts using Pearson’s correlation, which is summarized in the histogram plot shown in Figure 4E, and found low correlation in most of the transcripts.

Next, we performed differential RNA modification analysis of transcriptomes between ethanol and glucose growth using ELIGOS software. Based on the cut-off of odds ratios of ≥ 1.5 and adjPval < 0.01, from 349,015 sites in total, we identified 36,471 sites for respirofermentative (glucose-limited) growth and 3,817 sites differential sites for oxidative (ethanol) growth. The higher number of differential sites in the glucose-limited condition is along the line with the study of Tardu et al. (2019), who reported higher modified nucleotide fractions of yeast grown in glucose depravation conditions and lower modified nucleotide fractions of yeast grown in oxidative stress conditions by mass spectrometry analysis of yeast mRNA. The distribution of the identified differential sites per individual transcript is summarized in the histogram plot shown in Figure 4F. We observed that some known key metabolic genes, such as *ACC1*, *FAS2*, *ACS2*, *HMG1*, *PYC1*, and *PFK1*, have differential sites > 100 (see Supplementary Table S1). Zooming in at the central metabolic pathway shown in Figure 4G, we mapped relevant transcripts and their differential RNA modification sites to simultaneously assess the effect of transcriptional and posttranscriptional regulation during metabolic reprogramming required for the diauxic shift. The presented global overview shows the well-known adaptations (DeRisi et al., 1997) of yeast cells as they switch from glucose to ethanol by changing the gene expression of a number of key enzymes. In addition to transcriptional regulation, we found many transcripts that had undergone changes in base modifications under these conditions.

Genes under regulation to switch from glycolysis to ethanol utilization produce a very important metabolite acetyl-CoA, which as acetyl-CoA synthase has two isozymes, *ACS1* and *ACS2*. The main gene *ACS1* was transcriptionally upregulated in ethanol (indicated by a thick red box in Figure 4G); on the other hand, *ACS2* downregulated posttranscriptional modification (indicated by filled cyan color in Figure 4G). The posttranscriptional modification downregulation was also observed on key genes regulating the TCA cycle activity (*ACO1*, *ADH1*, *CIT*, *PYC1*, and *PYC2*), fatty acid biosynthesis *(ACC1*, *FAS1*, *FAS2*, and *HMG1*), and a gene involved in glycogen–trehalose homeostasis (*TPS1*). These results indicate that there exists a complex association between posttranscriptional modifications and metabolic reprogramming.

## Discussion

To study transcriptomes without application bias using ONT, we can perform either native or cDNA sequencing. Library preparations of native RNA sequencing has fewer steps, enabling a rapid characterization of RNA molecules as demonstrated in many studies (Jiang et al., 2019; Soneson et al., 2019; Wongsurawat et al., 2019; Radukic et al., 2020). However, direct sequencing of cDNA provides higher throughput data due to the faster chemistry speed, which is almost six times that of motor proteins. In our study, we encountered the problem of second-strand synthesis. This crucial step resulted in cDNA sequences with a high chimeric due to sequencing on single-strand DNA instead of double-strand, which is the optimized chemistry of ONT sequencing of DNA molecules.

The differential gene expression, which is a key result to study transcriptomes, derived from dRNA-seq and dcDNA-seq, is quite consistent at both the gene level and functional analysis level. Therefore, either method can be used to identify key transcriptionally regulated transcripts. Native RNA sequences provide opportunities to study posttranscriptional regulation, such as RNA methylations (Mendel et al., 2021; Yeager et al., 2021), leading to many efforts in the development of bioinformatics analysis to uncover accurate RNA modifications, as summarized in a review article by Furlan et al. (2021). However, dRNAseq has 3′ bias because the library preparations rely on the 3′ end adaptor ligation at the poly-A tail, leading to missed ligation of the broken pieces of RNA toward the 5′ end. This resulted in an incomplete picture of RNA modification throughout the transcript length, especially on the 5′ end of the transcript. Therefore, we need to improve the sample preparation, such as the 5′ race enrichment sequencing by ligating designed adaptors that are sequenced along with the transcript (Jiang et al., 2019; Ibrahim et al., 2021).

The RNA ribonucleotide modifications are known to be critical regulators of a wide range of biologically relevant processes. One of the unique advantages of dRNA-seq, which we have looked into here, is the ability to detect RNA ribonucleotide modifications directly, which cannot be accomplished by dcDNA-seq. However, the reads generated by dRNA-seq could contain higher error rates that are derived from modified bases. The summary of comparison between the two library preparation approaches is shown in Table 1.

**TABLE 1:** Comparison of dRNA-seq and dcDNA-seq.

	dRNA-seq	dcDNA-seq
Unique advantage	Retain the information of RNA modification	dcDNA reads were slightly longer
Accuracy of transcript	Not suitable because of modification signals leading to error	Higher accuracy
Input recommendations	500 ng (poly-A RNA)	250 ng (poly-A RNA)
Prep time	∼140 min	∼300 min
Cost	Less expensive than dcDNA-seq	More expensive because many enzymatic processes are required
Simple to perform	Yes	No
Limitations/difficulties	1. Require higher amount of poly-A RNA input	1. The problem of second-strand synthesis, possibly derived from an unsuccessful reaction
	2. Both hybridization and ligation of DNA adaptor and poly-A RNA are needed to continue to downstream library preparation steps (i.e., reverse transcription)	2. High fraction of chimeras leads to unmapped read
		3. Length of transcript sequence could be limited based on reserve transcriptase enzyme

In summary, our study showed the advantages and disadvantages of using dRNA-seq or dcDNA-seq to study transcriptomes in yeast. This will be useful information for research studies to select a method for transcriptional characterization in various research interests.

## Data Availability

The datasets presented in this study can be found in online repositories. The names of the repository/repositories and accession number(s) can be found at: https://www.ncbi.nlm.nih.gov/, PRJNA497103.
