# Supplementary material for: Native RNA or cDNA Sequencing for Transcriptomic Analysis: A Case Study on Saccharomyces cerevisiae
Source: Front Bioeng Biotechnol. 2022 Apr 12;10:842299. doi: 10.3389/fbioe.2022.842299 (PMC9039254; doi:10.3389/fbioe.2022.842299)
Supplement: Supplementary file 1 [file DataSheet3.PDF]

Data processing and mapping of reads

```
##Base calling dcDNA
guppy_basecaller -r -i fast5 -s fastq -c dna_r9.4.1_450bps_hac.cfg -x "cuda:0" --disable_pings --
qscore_filtering --min_qscore 7

##Base calling dcDNA
 guppy_basecaller -r -i fast5 -s fastq -c rna_r9.4.1_70bps_hac.cfg -x "cuda:0" --disable_pings --
qscore_filtering --min_qscore 7

##Filtering shot read < 200
NanoFil -l 200 fastq > fastq.200

## Alignment and index
minimap2 -ax splice -k14 -uf --eqx --secondary=no -t 50  referene.fasta fastq.200 | samtools sort
-o alignment.bam
samtools index alignment.bam
```

Evaluation of mRNA sequencing characteristics

In house script written in python can be found at https://gitlab.com/piroonj/eligos2/-/blob/master/Scripts/nanosummy.py?fbclid=IwAR1_LYxasQyx6LkiPuj_kC2K-l9ywaoR1NCd3GV1Xo4VopftppI3V_fNM6g

```
###Passing CIGAR string from nanosummy.py
nanosummy.py -t 8 --bam_summary --bam *.bam --data_matrix
```

Differential gene expression evaluation

```
## Generate count table
bedtools multicov -s -bams alignment.bam -bed yeast.annotation.gff3  > gene.count

##DESeq2
library(DESeq2)
count = read.delim('gene.count', header = F)
coll  <- data.frame(group =
as.factor(c(rep('ethD',3),rep('gluD',3),rep('ethR',3),rep('gluR',3))),rownames = colnames(count) )
dds <- DESeqDataSetFromMatrix(countData= count,colData = coll, design= ~ group)
dds <- DESeq(dds)

## extract statistical test
res.egD <- results(dds, contrast=c("group","ethD","gluD"),pAdjustMethod = "BH" )
write.table(res.egD , sep = '\t', file = 'Deseq_EvsG_dcDNA.txt')
res.egR <- results(dds, contrast=c("group","ethR","gluR"),pAdjustMethod = "BH" )
write.table(res.egR , sep = '\t', file = 'Deseq_EvsG_dRNA.txt')

##Piano
library(piano)
gsc = loadGSC('Yeast_GO.sif',type = 'sif')
library(snowfall)
```

```
cl <- makeCluster(4, type="SOCK")
p = res.egD$padj
rttest.DNA = runGSA(geneLevelStats = p,directions = fc,ncpus=4, geneSetStat =
'reporter',signifMethod ='nullDist',adjMethod = 'fdr',gsc =gsc)
Rttest.DNA = GSAsummaryTable(rttest.DNA ,save =T, file  = 'GOresults_res.DNA.txt')
p = res.egR$padj
fc =res.egR$log2FoldChange
rttest.RNA = runGSA(geneLevelStats = p,directions = fc,ncpus=4, geneSetStat =
'reporter',signifMethod ='nullDist',adjMethod = 'fdr',gsc =gsc)
Rttest.RNA = GSAsummaryTable(rttest.RNA  ,save =T, file  = 'GOresults_res.RNA.txt')
```

Inferring RNA modification from sequencing error profile

```
## Direct cDNA
fq=sample1.dcdna.fq
ref=reference.fa
bed=refgene.bed
threads=8
bname=sample_out

## Alignment
minimap2 -ax splice -k14 -uf --eqx --secondary=no -t ${threads} ${ref} ${fq} | samtools view -
Sb -F 3332 - > ${bname}.bam

## sort BAM
samtools sort -@${threads} -T ${bname}.bam.sort -o ${bname}.sorted.bam ${bname}.bam
samtools index ${bname}.sorted.bam

## variant calling
bcftools mpileup --threads ${threads} -Ou -f ${ref} ${bname}.sorted.bam | bcftools call --
threads 5 -mv -Ob -o ${bname}.sorted.bcf
bcftools index --threads ${threads} ${bname}.sorted.bcf

## direct RNA
fq=sample1.drna.fq
ref=reference.fa
bed=refgene.bed
threads=8
bname=sample_out

## Alignment
minimap2 -ax splice -k14 -uf --eqx --secondary=no -t ${threads} ${ref} ${fq} | samtools view -
Sb -F 3332 - > ${bname}.bam

## sort BAM
samtools sort -@${threads} -T ${bname}.bam.sort -o ${bname}.sorted.bam ${bname}.bam
samtools index ${bname}.sorted.bam
```

Filter out mapped reads shorter than 200 bases
eligos2 map_preprocess -aln 200 -i ${bname}.clean.bam

Run ELIGOS
eligos2 rna_mod --threads ${threads} --esb 0 --oddR 1.2 -i ${bname}.clean.bam -reg ${bed} -ref ${reference} -o ${bname}.eligos_results

Run multi_samples_test (use example from GitLab)

https://gitlab.com/piroonj/eligos2#4-identification-of-rna-modifications-from-replicates-using-the-cochran-mantel-haenszel-cmh-test
eligos2 multi_samples_test --test_mods 4.example_YPL061W_baseExt0/drna_ethanol_*.txt --ctrl_mods 4.example_YPL061W_baseExt0/drna_glucose_*.txt --prefix ethanol_vs_glucose

RNA structure prediction using ShaKer and RNAplfold

```
## Run ShaKer
import sys
import shaker.rna_tools.rna_io as rio
import shaker.simushape as sim
from shaker.rna_tools.rnaplfold import rnaplfold
from Bio import SeqIO
def cal_acc_shaker(model, seq):
prediction = (seq, sim.predict(model, seq))
return rnaplfold(*prediction)
def main(rna_fasta, output_file):
data = rio.get_all_data("ShaKer/data/RNA16.react", "ShaKer/data/RNA16.dbn")
model = sim.make_model(data,data.keys())
w_f = open(output_file, 'w')
for counter, record in enumerate(SeqIO.parse(rna_fasta, 'fasta'), start=1):
if counter % 10:
print(counter)
seq_id = record.id
seq = str(record.seq).upper()
str_acc_shaker = ""
try:
list_acc_shaker = cal_acc_shaker(model, seq)
str_acc_shaker = ','.join("{:.6f}".format(x) for x in list_acc_shaker)
except:
pass
write_line = "{}\t{}\t{}\t{}\n".format(seq_id, len(seq), seq, str_acc_shaker)
w_f.write(write_line)
w_f.close()
if __name__ == "__main__":
rna_fasta = sys.argv[1]
output_file = sys.argv[2]
main(rna_fasta, output_file)
```

Genomic locations of loci and transcripts comparison

Genomics loci comparision
bedtools intersect -s -a bedA.bed -b bedB.bed
