# Supplementary figures and images for: Native RNA or cDNA Sequencing for Transcriptomic Analysis: A Case Study on Saccharomyces cerevisiae
Source: Front Bioeng Biotechnol. 2022 Apr 12;10:842299. doi: 10.3389/fbioe.2022.842299 (PMC9039254; doi:10.3389/fbioe.2022.842299)

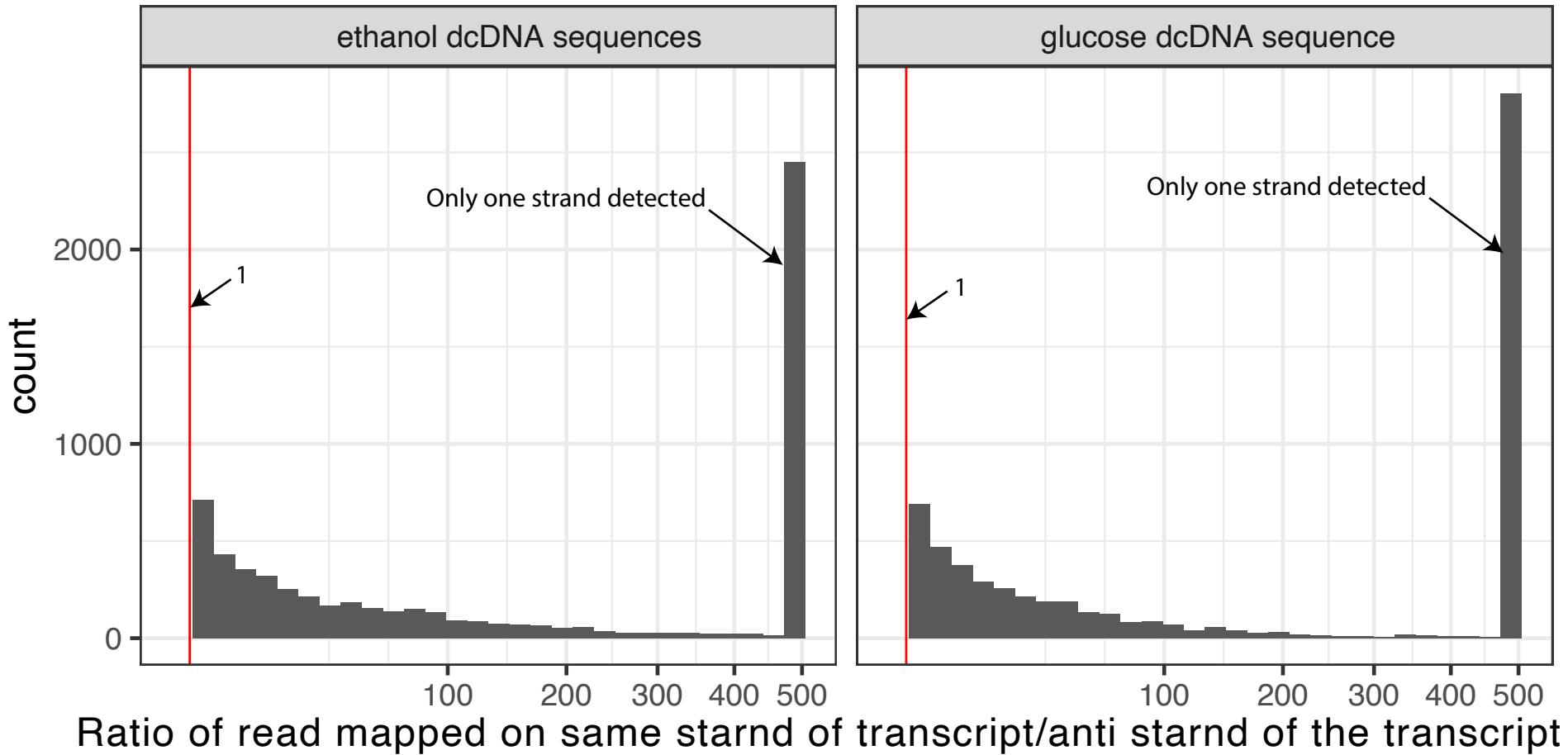

Supplement: Supplementary file 2 [file DataSheet1.PDF]
